# Biocontrol of *Botrytis cinerea* on Grape Berries as Influenced by Temperature and Humidity

**DOI:** 10.3389/fpls.2020.01232

**Published:** 2020-08-14

**Authors:** Giorgia Fedele, Chiara Brischetto, Vittorio Rossi

**Affiliations:** Department of Sustainable Crop Production (DI.PRO.VE.S.), Università Cattolica del Sacro Cuore, Piacenza, Italy

**Keywords:** gray mold, biological control, biological control agents, integrated pest management, modeling, *Vitis vinifera*

## Abstract

Six commercial biocontrol agents (BCAs, containing *Aureobasidium pullulans, Bacillus amyloliquefaciens, Bacillus amyloliquefaciens plantarum, Bacillus subtilis, Pythium oligandrum*, or *Trichoderma atroviride*) were applied to ripening berries that were then incubated at one of four temperatures (T, 15, 20, 25, and 30°C) and one of four relative humidity levels (RH, 60, 80, 90, and 100%). After 1 to 13 days of incubation (BCA colonization period), the berries were inoculated with conidia of *Botrytis cinerea* and kept at 25°C and 100% RH for 7 days, at which time Botrytis bunch rot (BBR) was assessed. The response of BBR control to T/RH conditions and BCA colonization period differed among BCAs; the coefficients of variation among the BCAs ranged from 44.7 to 72.4%. An equation was developed that accounted for the combined effects of T, RH, and BCA colonization period on BBR control. The equation, which had an R^2^>0.94, could help farmers select the BCA to be used for a specific application based on weather conditions at the time of treatment and in the following days.

## Introduction

The necrotrophic fungal pathogen *Botrytis cinerea* Pers. Fr. [teleomorph *Botryotinia fuckeliana* (de Bary) Whetzel] causes Botrytis bunch rot (BBR), which is a major disease of grapevines (Elad et al., 2016). Although *B. cinerea* can damage all vine organs, its infection of ripening berries results in especially large losses in quantity and quality (Jarvis, 1977; Williamson et al., 2007; Elad et al., 2016). Because of physiological changes in ripening berries (Kretschmer et al., 2007; Mundy and Beresford, 2007; Deytieux-Belleau et al., 2009) but also because environmental conditions during the latter part of the growing season tend to favor the infection (Nair et al., 1988; Ciliberti et al., 2015), the susceptibility of berries to *B. cinerea* increases with maturity (Deytieux-Belleau et al., 2009), from veraison to harvest (from growth stage (GS) 81 to GS<89 of Lorenz et al., 1995). Botrytis bunch rot on ripening berries can result from i) latent infections established during flowering, ii) direct berry infection caused by conidia, and iii) berry-to-berry infection caused by mycelium originating from previously infected berries within the cluster (Elmer and Michailides, 2007; González-Domínguez et al., 2015). The disease on ripening berries is traditionally controlled by repeated fungicide applications (González-Domínguez et al., 2019).

In recent years, use of fungicides during ripening has been subjected to increasing limitations in order to reduce or eventually eliminate chemical residues on grapes and in wines (Verger and Boobis, 2013). Negative effects on the environment and the resistance of *B. cinerea* populations to most chemical fungicides (Leroux, 2007; Fernández-Ortuño et al., 2016) also contribute to an increasing interest in alternatives to chemical fungicides for BBR management (Harman, 2000; Elad and Freeman, 2002; Elmer et al., 2005; Tracy, 2014).

One promising alternative for BBR management is the use of microorganisms as biocontrol agents (BCAs) (Pertot et al., 2017; Fedele et al., 2020c; Fedele et al., 2020d). BCAs may suppress *B. cinerea via* various modes of action, including competition for nutrients and space, antibiosis, parasitism, and induced host plant resistance (Elad and Freeman, 2002; Elmer and Reglinski, 2006; Haidar et al., 2016).

In spite of the extensive research on BCAs (O’Neill et al., 1996; Schoene, 2002; Jacometti et al., 2010; Calvo-Garrido et al., 2014; Pertot et al., 2017; Calvo-Garrido et al., 2018; Escribano-Viana et al., 2018; Rotolo et al., 2018; Calvo-Garrido et al., 2019; Fedele et al., 2020a), only a few commercial products containing BCAs are available in Europe (Nicot et al., 2016), where farmers still mainly rely on chemical fungicides for BBR control. The limited use of BCAs for BBR control may be related to their inconsistent efficacy across seasons or across local agronomic conditions (Tracy, 2014; Calvo-Garrido et al., 2019), i.e., multiple factors and complex processes generally determine BCA efficacy (Rosenheim et al., 1995). This complexity should be expected given that BCAs are living organisms that dynamically interact with the target pathogen, the host plant, and the microbial communities on the host surfaces in a changing physical environment (Fedele et al., 2020c). Fluctuating environmental conditions, in particular, strongly influence BCA survival, establishment, growth, and efficacy (Elad and Freeman, 2002; Kredics et al., 2003; Xu et al., 2010; Fedele et al., 2020c).

It follows that the successful integration of BCAs into a disease management strategy requires an understanding of their environmental requirements. Temperature, humidity, and other environmental conditions have been evaluated as key factors determining BCA efficacy (Mitchell et al., 1987; Jackson et al., 1991; Elad et al., 1993; Hannusch and Boland, 1996; Dik et al., 1999; Kredics et al., 2003; Fedele et al., 2020c), but the complex relationships between BCAs and multiple environmental factors remain poorly understood (Deacon and Berry, 1993; Whipps, 1997).

Fedele et al. (2020a) recently used a general model to study how control of BBR by a theoretical BCA is affected by the control mechanism, timing of BCA application, and environmental conditions. The authors found that temperature and moisture conditions affecting the growth and survival of the theoretical BCA were more important than its mechanism of control or the timing of its application. The latter study indicated that additional research is needed to determine how the efficacies of specific BCAs of *B. cinerea* are affected by fluctuating temperatures and humidity.

In the current research, we studied the effect of six commercial biocontrol products (each with different BCA) on the development of BBR on grape berries. Although conducted in growth chambers, the experiment used four temperature regimes and four relative humidity regimes that simulated a range of vineyard conditions. The basic experimental scenario involved the transfer of individual berries (at different stages of maturity) from a vineyard into growth chambers, where the berries were treated with the BCAs. After the BCAs had time to develop on the berry surfaces (1 to 13 days; this was another experimental variable) under different environmental conditions, the berries were inoculated with conidia of *B. cinerea*. The berries were subsequently kept under optimal conditions for disease development before they were assessed for BBR.

## Materials and Methods

### Plant Material

Berries were collected in 2018 and 2019 in an 11-year-old vineyard (in 2018) located at Castell’Arquato (44°51′26.1′′N 9°51′20.7′′E, 400 m a.s.l.) in the Emilia-Romagna region. The vineyard was planted with cv. Merlot, and the vines were trained using a Guyot system; the within- and between-row spacing were 1.0 and 2.3 m, respectively. Powdery and downy mildews were controlled according to an integrated pest management program (Rossi et al., 2012), and the fungicides applied were not effective against *B. cinerea*.

Ripening berries were collected (with their pedicels) in 2018 and 2019 on the following dates: 03, 10, and 17 September in 2018; 26 August and 2 and 9 September in 2019. The ripening stages of the berries at sampling were measured as degrees Babo (an indicator of sugar content), which were 19.3, 20.0, and 20.3 in 2018, and 16.4, 18.6, and 19.0 in 2019.

Berries were transported to the laboratory in a cooler, where they were rinsed under tap water for 3 min, disinfested with 2/3 of distilled water and 1/3 of 5% sodium hypochlorite to remove epiphytic microflora, and finally rinsed again with sterile water.

### Treatment of Berries With Biocontrol Agents

Six commercial BCAs were used (**Table 1**). These products were dispersed in double-distilled sterile water (pH 6.5) at the label dose, and berries were treated by immersion. The viability of the BCAs was confirmed by plating the product suspensions on PDA (potato dextrose agar, Biolife Italiana S.r.l., Milano, Italy). Non-treated (NT) berries were used as the control, and these were immersed in double-distilled, sterile water.

**Table 1:** Biocontrol agents (BCAs) used in the experiment.

Active ingredient	Commercial product (acronym)	Producer	Label dose (g/ha)
*Bacillus amyloliquefaciens* D747	Amylo-X (AMY)	CBC S.r.l.	2000
*Aureobasidium pullulans* DMS 14941-14940	Botector (BOT)	Manica S.p.A.	400
*Pythium oligandrum* M1	Polyversum (POL)	Gowan Italia S.r.l.	200
*Bacillus subtilis* QST 713	Serenade max (SER)	Bayer S.p.A.	3,000
*Bacillus amyloliquefaciens* FZB24	Taegro (TAE)	Syngenta	370
*Trichoderma atroviride* SC1	Vintec (VIN)	Belchim S.p.A.	1,000

Berries were then placed in metal boxes (20 × 15 cm) over a metal grid net so that berries did not touch each other or the box bottom; there were 15 berries per box. After treatment, boxes were incubated in growth chambers with a 12-h photoperiod and at different regimes of temperature (15, 20, 25, and 30°C) and relative humidity (60, 80, 90, and 100% RH). Different RH values were obtained by placing 200 ml of different glycerol/double-distilled water solutions (Dallyn and Fox, 1980) or double-distilled water on the bottom of the metal boxes. The true RH inside the boxes was checked with a data logger (Tinytag Plus 2, Gemini Data Loggers, Chichester, England). There were three boxes per each T/RH regime. The experiment was performed three times per year for 2 years, with berries sampled at different ripening stages.

### Inoculation of Berries With *Botrytis cinerea*

At 1, 3, 6, 9, or 13 days after treatment with BCAs, the berries in each box were inoculated with a conidial suspension of *B. cinerea*, isolate 213T, which belongs to the *transposa* sub-species and is highly aggressive (Ciliberti et al., 2016). The conidia were obtained from 10-day-old cultures grown on PDA at 20°C and with a 12-h photoperiod using white and near-UV (UV-A at 370 nm) light (Black Light UV-A, L18 w/73, OSRAM, Munich, Germany). The conidial suspensions were prepared by flooding the dishes with sterile-distilled water and gently scraping the agar surface with a sterile rod. The suspension was passed through two layers of sterilized gauze (autoclaved at 120°C for 20 min), and the conidia were enumerated with a hemocytometer. The concentration of conidia was adjusted to 10^5^/ml by adding double-distilled sterile water. The conidial suspension was uniformly distributed on the berries by using a hand sprayer, 1 ml of suspension per box.

The boxes were then placed in growth chambers at 25°C and with 100% RH and a 12-h photoperiod for 7 days to promote the germination of *B. cinerea* conidia and the development of BBR.

### Disease Assessment

One week after the single berries were inoculated with *B. cinerea*, BBR severity was assessed as the percentage of the surface of each berry with BBR symptoms; the severity of the entire replicate (i.e., of the 15 berries in each box) was then determined by using the standard area diagram of Hill et al. (2010).

### Data Analysis

BBR severity data were subjected to a factorial analysis of variance (ANOVA), in which the factors were BCA treatment (six BCAs plus the untreated control, NT), T/RH regime (combinations of T and RH), and the number of days that berries were incubated before they were inoculated with *B. cinerea* (BCA colonization period: 1, 3, 6, 9, or 13 days). The experimental design was a split-split, with BCAs as the main plots, T/RH regimes as the split plots, and BCA colonization periods as the split-split plots. The 2 years and the different ripening stages at which berries were sampled were considered blocking factors; according to statistical theory and experimental design, a blocking factor is a source of variability that is not of primary interest for the experiment (Quinn and Keough, 2002). The BBR severity data (in %) were subjected to arcsin transformation before the ANOVA to make variances homogeneous.

To assess the variability in BBR severity as affected by T/RH regime and colonization period within each BCA, coefficients of variation (CV, in %), or relative standard deviations (SDs) were calculated as the ratio of the SD to the mean; the higher the CV, the greater the variability generated by T/RH regimes and the length of the BCA colonization period.

To further investigate the variability generated by T/RH regimes and length of colonization period within each BCA, BBR severity data were expressed as disease control relative to NT as follows: the difference between BBR severity on the untreated berries (i.e., the berries that were not treated with any BCA before they were inoculated with *B. cinerea*) and treated berries (i.e., the berries that were treated with a BCA before they were inoculated with *B. cinerea*) was calculated and divided by the maximal difference found in the experiment for the specific BCA. As a hypothetical example, consider berries that were untreated or treated with BCA X and then were incubated at 15°C and 100% RH for 6 days before being inoculated with *B. cinerea*; in this first hypothetical example, BBR severity was 30% for untreated berries and 5% for treated berries, resulting in a 25% difference. As a second hypothetical example, consider berries that were incubated at 25°C and 100% RH for 9 days before *B. cinerea* inoculation and that had a BBR severity of 55% if untreated and 10% if treated with BCA X, resulting in a 45% difference, which was the greatest difference for BCA X among all combinations of T, RH, and length of colonization period. Therefore, the relative control provided by BCA X was 25/45 = 0.55 for the first example and 45/45 = 1 for the second example. As indicated by these hypothetical examples, relative control is scaled between zero (no control, i.e., no difference with respect to NT) and 1.0 (highest control, i.e., the widest difference with respect to NT). Data were rescaled as described because the aim was to evaluate the within-BCA variability in the control of BBR under different temperature and relative humidity regimes rather than to compare the efficacy of the different BCAs.

The relative control data (Y) were then fit to the following equation:

[1] $$Y=(\text{α}Teq^{\text{β}}{\left(1-\text{Teq}\right)}^{\phi })\;(1-\varphi )(1-{\text{λ}}^{\text{VPD}}))\;(1-{\text{ς}}^{\text{dbi}})$$

when: Y<0, Y=0; Y>1, Y=1

where α, β, ϕ, φ, λ, and ς are the equation parameters; Teq are equivalents of temperature, defined as Teq=(T-Tmin)/(Tmax-Tmin), where T is the temperature regime (in °C), and Tmin and Tmax are cardinal temperatures (in °C); dbi is the number of days between the application of BCA and inoculation with *B. cinerea*; and vapor pressure deficit (VPD) (in kPa) is the vapor pressure deficit calculated as follows (Buck, 1981):

VPD=0.61121 exp((18.678-T/234.5)(T/(257.14+T)))(1-RH/100)

where RH is relative humidity (in %).

Equation [1] was selected among a set of candidate equations by using the Akaike information criterion (AIC), which is an estimator of the relative quality of statistical models for a given set of data (Akaike, 1981). Equation parameters, Tmin, and Tmax were estimated using the non-linear regression procedure of SPSS software (IBM SPSS Statistics, version 25), which minimizes the residual sums of squares using the Marquardt algorithm.

In equation [1], the first term, [α Teq^β^ (1-Teq)^ϕ^], accounts for the effect of temperature according to the bell-shaped curve of Analytis (Analytis, 1977), with parameters α, β, and ϕ defining the top, symmetry, and size of the curve, respectively. The second term, [1-φ (1-λ^VPD^)], accounts for the combined effect of T and RH on Y according to an asymptotic equation, where 1 is the maximum attainable value for Y, φ is the value for Y at VPD=0, and λ is proportional to the relative rate of decrease for Y when VPD increases. The third term, (1-ς^dbi^), accounts for the effect on Y of the time elapsed from the BCA application, where 1 is the maximum attainable value for Y and ς is proportional to the relative rate of increase for Y when time elapses.

Goodness-of-fit of equation [1] to the relative control data for the six BCAs was assessed for the whole dataset based on the adjusted R^2^ (which was estimated by conducting a linear regression between the observed and the predicted values), the root mean square error (RMSE), the coefficient of residual mass (CRM), and the concordance correlation coefficient (CCC) (Nash and Sutcliffe, 1970; Lin, 1989). In brief, RMSE represents the average distance of real data from the fitted line, and CRM is a measure of the tendency of the equation to overestimate or underestimate the observed values (a negative CRM indicates a tendency of the model toward overestimation) (Nash and Sutcliffe, 1970). CCC is the product of two terms: the Pearson correlation coefficient and the coefficient Cb, which indicates the difference between the best fitting line and the perfect agreement line (CCC = 1 means perfect agreement) (Madden et al., 2007).

## Results

Overall BBR severity on untreated berries was 29.5±0.85%. In both years, BBR severity increased with sampling date, i.e., increased as berries ripened: BBR severity was 42.1, 56.5, and 59.7% in 2018, and was 21.2, 22.6, and 24.3% in 2019. Because this source of variability was not of primary interest in this study, it was considered a blocking factor in the ANOVA.

The ANOVA showed a significant effect of the main factors BCA, T/RH regime, and BCA colonization period (all at P<0.001), which explained 17.5, 42.4, and 4.1%, respectively, of the total variance in BBR severity. The interactions BCA × T/RH regime and BCA × BCA colonization period were also significant (P=0.009 and P=0.006, respectively), and accounted for 9.7 and 6.5% of the total variance in BBR severity, respectively. The interactions T/RH regime × BCA colonization period and BCA × T/RH regime × BCA colonization period were not significant (P>0.05 in both cases), and together accounted for 29.7% of total variance in BBR severity. These data show that the number of days that BCAs grew on the surface of berries before *B. cinerea* inoculation and also the T/RH regime during this period significantly affected BBR severity but that the response of each BCA to T/RH regime and colonization period differed among BCAs.

**Figure 1:**
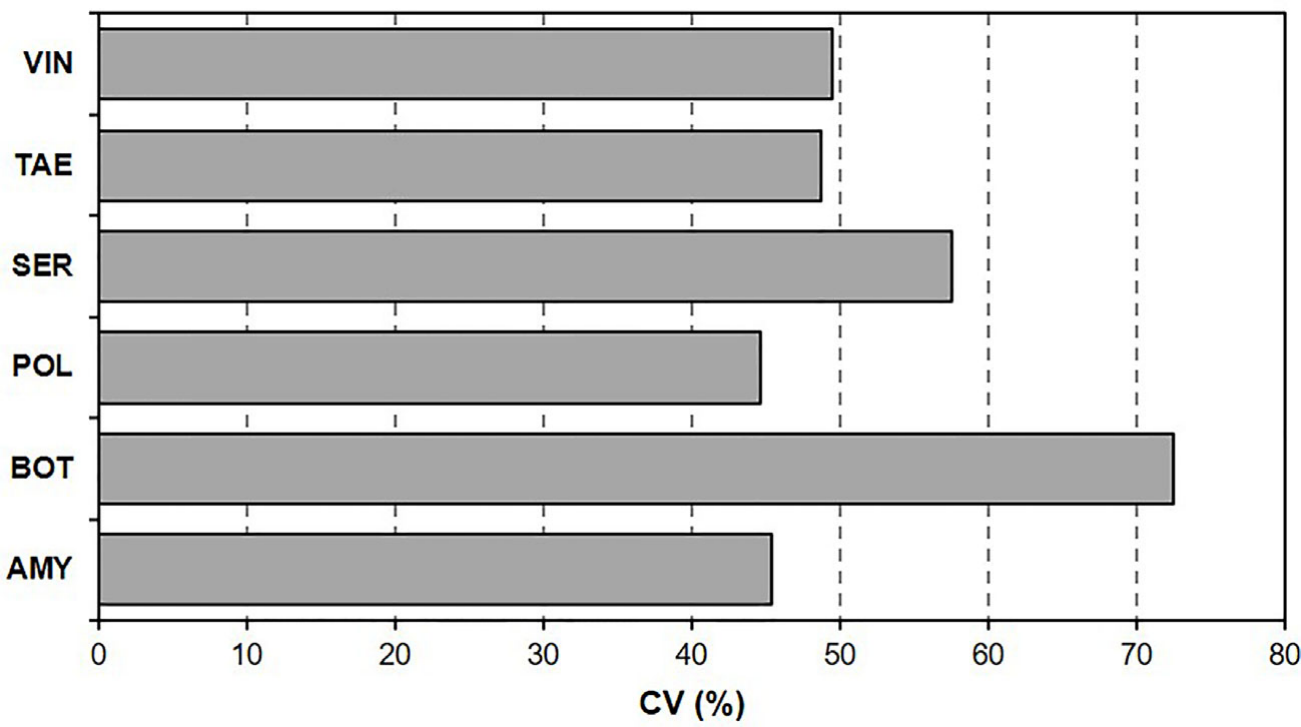
Coefficient of variation (CV, %) of Botrytis bunch rot (BBR) severity on grape berries. After the berries were treated with six biocontrol agents (BCAs), they were subjected to different temperature and relative humidity regimes for different BCA colonization periods before they were inoculated with *Botrytis cinerea* and kept under conditions that were optimal for BBR.

The coefficient of variation (**Figure 1**) of BBR severity data ranged from 44.7% [for polyversum (POL)] to 72.4% [for botector (BOT)], which indicated that some BCAs were more sensitive than other BCAs to T/RH regimes and the length of the BCA colonization period before *B. cinerea* inoculation.

**Figure 2:**
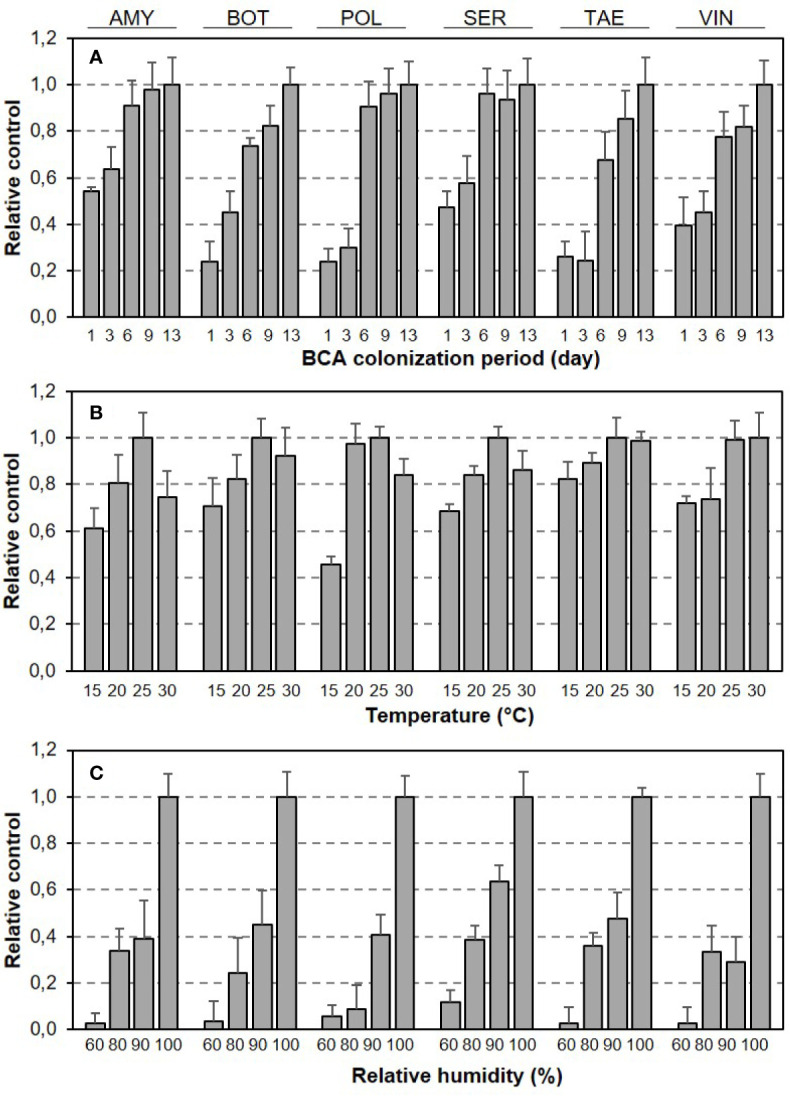
Relative control of Botrytis bunch rot severity on grape berries by six biocontrol agents (BCAs) as affected by **(A)** the length of colonization period (in days) before the BCA-treated berries were subsequently inoculated with *Botrytis cinerea*, and by **(B)** the temperature and **(C)** relative humidity that the berries were subjected to in the period between BCA treatment and *B. cinerea* inoculation. Bars are means (+ SE) of different T/RH regimes in **(A)** and of different numbers of days between BCA treatment *B. cinerea* and inoculation in **(B, C)**.

Relative BBR control by BCAs as affected by length of BCA colonization period and T/RH regime is shown in **Figure 2**. Although the dynamics of relative control over time were generally similar for the six BCAs, the BCAs differed in the time required to achieve maximal control, i.e., some BCAs were faster than others (**Figure 2A**). Although the responses of BBR control to T and RH (both were considered as main effects) had bell-shaped and exponential patterns, respectively, for all of the BCAs, the BCAs differed in the size of response to specific T or RH conditions (**Figures 2B, C**).

Equation [1] provided a good fit for the relative control data for all of the BCAs, with R^2^>0.94, RMSE≤0.08, CRM≤0.01, and CCC>0.97 (**Table 2**). This indicated that solving equation [1] for any time period between the application of a BCA and the occurrence of a *B. cinerea* infection (in the interval 1 to 13 days), and for any combination of T (between 15 and 30°C) and RH (between 60 to 100%), provides a reliable prediction of the relative control of BBR by the treatment.

**Table 2:** Parameters of equation [1] for the six biocontrol agents (BCAs), and statistics for goodness-of-fit to real data.

BCA	Cardinal temperatures^1^		Equation parameters^2^						Statistics^3^			
	Tmin	Tmax	α	β	ϕ	φ	λ	ς	R^2^	RMSE	CRM	CCC
AMY	10	35	2.626	0.846	0.647	0.11	1.05	0.622	0.957	0.073	0.007	0.977
BOT	5	40	2.449	0.873	0.532	0.10	1.00	0.805	0.992	0.031	0.002	0.996
POL	10	38	6.067	1.363	1.217	0.025	0.95	0.786	0.963	0.076	0.010	0.979
SER	10	38	2.051	0.593	0.516	0.17	0.95	0.669	0.967	0.061	0.008	0.983
TAE	5	40	1.817	0.540	0.340	0.19	1.08	0.834	0.966	0.068	0.003	0.982
VIN	5	38	2.657	0.973	0.516	0.09	1.02	0.783	0.949	0.080	0.006	0.974

^1^ Estimates of Tmin and Tmax used for the calculation of equivalents of temperature, Teq, used in equation [1]; ^2^ estimates of parameters of equation [1]: Y = [α Teq^β^ (1-Teq)^ϕ^) (1-φ (1-λ^VPD^)] (1-ς^dbi^); ^3^ adjusted R^2^, root mean square error (RMSE), coefficient of residual mass (CRM), concordance correlation coefficient (CCC).

**Figure 3:**
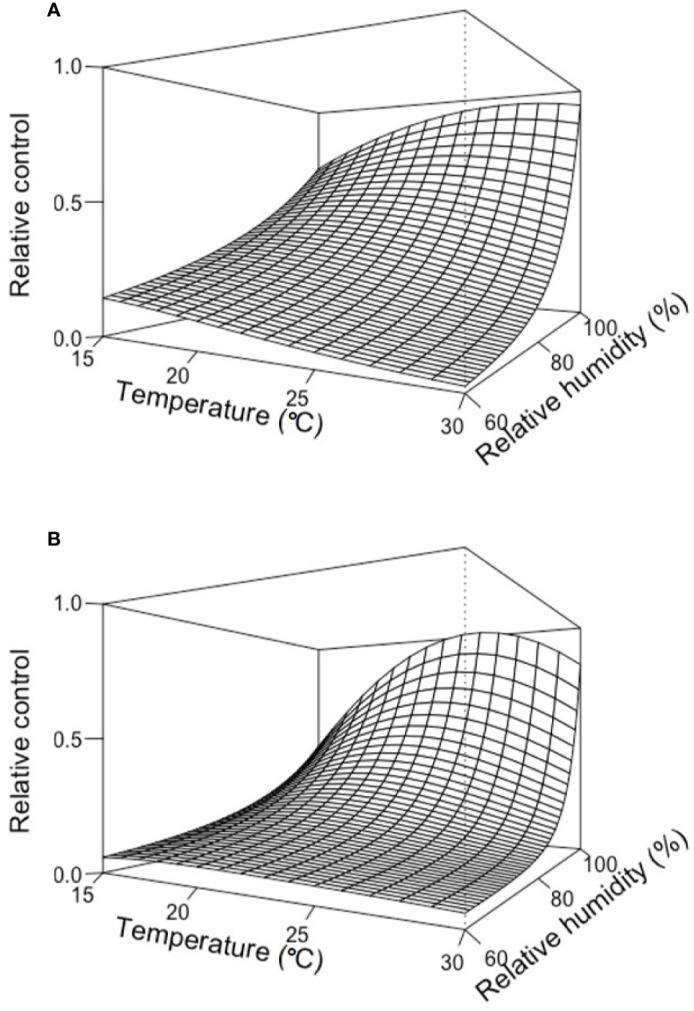
Relative control of Botrytis bunch rot on grape berries that were treated with two biocontrol agents (BCAs) (BOT in **A** and POL in **B**; see **Table 1**) and then incubated for 13 days (dbi = 13) at different temperature (T) and relative humidity (RH) values before they were inoculated with *Botrytis cinerea*. Following inoculation with *B. cinerea*, the berries were kept under T and RH conditions that favored disease development (25°C and 100% RH). The plots were generated with equation [1] and the parameters listed in **Table 2**.

**Figure 3** provides two examples of the three-dimension plot of equation [1] with the parameters of **Table 2** for BOT and POL, with dbi=13 days.

**Figure 4:**
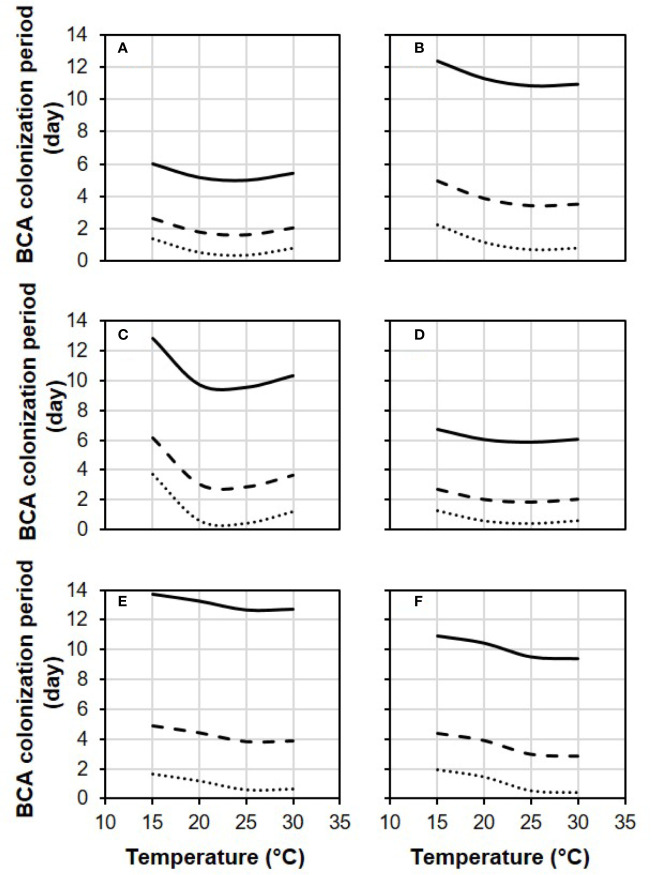
Effect of temperature on the length of the biocontrol agent (BCA) colonization period required by the six BCAs (**A**: AMY; **B**: BOT; **C**: POL; **D**: SER; **E**: TAE; **F**: VIN, see **Table 1**) to attain 0.1 (dotted line), 0.5 (dashed line), and 0.9 (solid line) relative control of Botrytis bunch rot. The colonization period is the number of days between treatment of berries with a BCA and inoculation of berries with *Botrytis cinerea*. Berries were treated with the BCAs listed in **Table 1** and were then incubated in growth chambers at different temperatures and with different RH values (values in this figure are for RH = 100%). After 1, 3, 6, 9, and 13 days, berries were inoculated with *B. cinerea* and incubated at 25°C and 100% RH, which favored disease development. Lines were drawn by using the equation [1] and the parameters listed in **Table 2**.

In **Figure 4**, equation [1] was used to calculate the colonization period (in days) required by each BCA to provide 0.1, 0.5, and 0.9 relative control of BBR at different temperatures and with RH=100%. Some BCAs, like AMY and POL (**Figures 4A, C**), required shorter colonization period than others before *B. cinerea* inoculation to attain a specific level of control, and the length of this period was temperature-dependent. To provide 0.5 relative BBR control, for instance, both POL (**Figure 4C**) and VIN (**Figure 4E**) required fewer than 3 days at 25°C but required 6.0 and 4.3 days, respectively, at 15°C.

**Figure 5:**
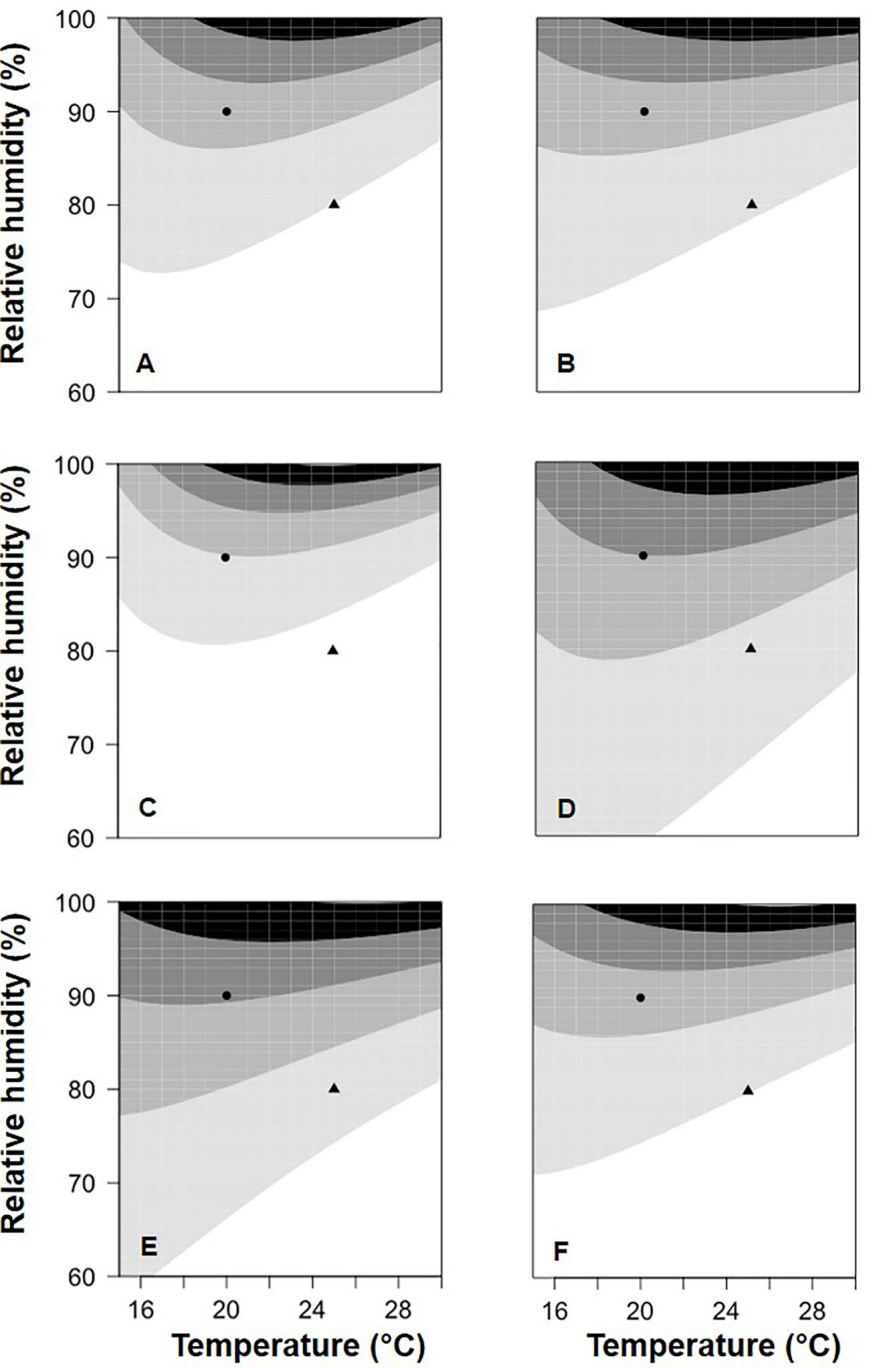
Relative control of Botrytis bunch rot severity on grape berries that were treated with six biocontrol agents (BCAs) (**A**: AMY; **B**: BOT; **C**: POL; **D**: SER; **E**: TAE; **F**: VIN, see **Table 1**) and then incubated at different temperatures (T) and with different relative humidity (RH) values. After 13 days (for the data in this figure), berries were inoculated with *Botrytis cinerea* and then incubated at 25°C and 100% RH, which favored disease development. The contour plots (which are based on the 3-dimensional graphs in Figure 3 with BCA colonization period of 13 days) identify five areas of relative control of berry bunch rot by the six BCAs: L (low, 0 to 0.2 relative control; the white area); ML (medium-low, 0.2 to 0.4; the light gray area); M (medium, 0.4 to 0.6; the medium gray area); MH (medium-high, 0.6 to 0.8; the dark gray area); and H (high: 0.8 to 1; the black area).

In **Figure 5**, equation [1] was used to calculate the relative BBR control provided by the application of the six BCAs with a colonization period of 13 days between BCA treatment and *B. cinerea* inoculation; in **Figure 5**, relative control values were grouped into five categories, from low to high values. The BCAs differed in their response to T/RH, i.e., some provided higher control than others at specific combinations of temperature and relative humidity. With the combination T=20°C and RH=90% (the circles in **Figure 5**), for example, relative control was medium–low for BOT (**Figure 5B**), medium for AMY (**Figure 5A**) and VIN (**Figure 5F**), between medium and medium–high for POL (**Figure 5C**) and TAE (**Figure 5E**), and medium–high for SER (**Figure 5D**). In another example, i.e. with T=25°C and RH=80% (the triangles in Fig. 5), relative control was low for BOT (**Figure 5B**) and VIN (**Figure 5F**), and was medium–low for the others.

## Discussion

In this research, we studied six commercial BCAs that are currently available for the control of *B. cinerea* in vineyards and that differ in their modes of action. *Bacillus amyloliquefaciens* subsp. *plantarum* strain D747 (AMY), *Bacillus subtilis* strain QST 713 (SER), and *B. amyloliquefaciens* strain FZB24 (TAE) are spore-forming bacteria whose major mode of action is antibiosis (Leifert et al., 1995; Borriss, 2015). *Aureobasidium pullulans* strains DMS 14941-14940 (BOT) are common fungal epiphytes of grapevines that can colonize wounds, produce hydrolytic enzymes, and form a biofilm (Parafati et al., 2015), and that can also produce volatile organic compounds that prevent the germination of conidia (Di Francesco et al., 2015). *Pythium oligandrum* strain M1 (POL) is a mycoparasitic oomycete that attacks the host fungus by lysis or penetration of hyphae (Laing and Deacon, 1991). Like other *Trichoderma* spp., *T. atroviride* strain SC1 (VIN) has multiple modes of action, including induction of plant resistance, mycoparasitism, antibiosis, and competition for space and nutrients (Pertot et al., 2013). All of these BCAs have been proved to provide control of BBR under vineyard conditions, although with variable efficacy (Pertot et al., 2017; Alessandri et al., 2018; Rotolo et al., 2018; Calvo-Garrido et al., 2019).

In this research, rather than obtain additional data on the efficacy of these BCAs in controlling BBR in vineyards, we assessed the effect of temperature and humidity on their efficacy. For this reason, we expressed data on BBR control in terms of relative control, i.e., as a proportion of the highest level of control provided by each BCA under the most favorable T/RH regime. The use of relative control enabled us to determine the best T/RH conditions for each BCA and to compare the T/RH responses among the six BCAs.

We conducted an experiment under controlled environmental conditions, in which grape berries were treated with the BCAs and inoculated with conidia of *B. cinerea* at different times after BCA treatment. Artificial inoculation under controlled environmental conditions has been frequently used to study relationships among microbial agents and target pathogens. Guetsky et al. (2001), for example, determined the effects of a separate or combined application of *Pichia guilliermondii* and *Bacillus mycoides* on *B. cinerea* spore germination, lesion formation, and lesion development on strawberry leaves under different combinations of T/RH. In another study, Hannusch and Boland (1996) examined the interactions between *B. cinerea* and seven BCAs under controlled environmental conditions to determine the influence of T/RH regimes on gray mold of bean. This approach makes possible to vary and study some factors (like T, RH, and length of BCA colonization period) while others that act under natural conditions are kept constant. For instance, disinfestation of berry surfaces eliminates epiphytic microbial populations that could differ among seasons, vineyards, and position on the vine and that could interact with BCAs (Zhang et al., 2008; Massart et al., 2015). Artificial inoculation also removed the effect of varying inoculum dose and time of *B. cinerea* infection, which may influence BBR development (Coertze et al., 1999) and therefore BCA efficacy.

BBR development in the untreated control was influenced by the ripening stage of the inoculated berries, i.e., disease severity increased as ripening advanced. This result was expected because the susceptibility of berries to infection by *B. cinerea* increases during ripening due to the increase in sugar and yeast assimilable nitrogen concentration, the changes in the phenolic compounds in the skin cell walls, and the decrease in water activity at the fruit surface (Kretschmer et al., 2007; Mundy and Beresford, 2007; Deytieux-Belleau et al., 2009). Because the effect of ripening stage was not a focus of this research, it was considered a blocking factor, i.e., a variable that is of no interest but that affects BBR and is controlled for, thus leading to greater accuracy in the statistical analysis (Quinn and Keough, 2002). As a consequence, the relative control provided by BCAs was not biased by the ripening stage of the collected berries.

The response to T/RH differed among BCAs and was also affected by the length of the BCA colonization period, i.e., the number of days between BCA treatment and *B. cinerea* inoculation. Some of the BCAs were more influenced by different environmental conditions than others, as demonstrated by the different coefficient of variation shown in **Figure 1**. That environmental conditions are able to influence the survival, growth, and efficacy of BCAs against *B. cinerea* has been abundantly demonstrated (see the recent review of Fedele et al., 2020b). A wide range of BCAs have been reported to control *B. cinerea* under controlled laboratory conditions and mostly with *in vitro* experiments, and only few have been tested with *in planta* studies (Nicot, 2011). Overall, few studies have provided information about the effects of both T and RH on BCA efficacy [against *B. cinerea*] (Fedele et al., 2020c) and, to our knowledge, no study similar to the current one has been previously reported.

The different responses of BCAs to T/RH conditions may help explain the variability reported in other studies that applied BCAs in different vineyards, different regions, or different years (O’Neill et al., 1996; Nicot, 2011; Abbey et al., 2018). Calvo-Garrido et al. (2019), for example, recently reported that the reduction in BBR severity resulting from treatment with *B. subtilis* (SER) was 54 and 17% at two locations in 2015, 21% and −43% at two locations in 2016, and 46% at two locations in 2017 (pooled data). In one of these vineyards, SER provided better control than *A. pullulans* (BOT) in 2015 (54 *vs.* 46%) but the opposite was found in 2016 (21 *vs.* 37%). Because the materials and methods used by Calvo-Garrido et al. (2019) were consistent among years and vineyards, differences in the efficacy of SER and BOT in different seasons may be explained by different responses to weather conditions. Ojiambo and Scherm (2006) highlighted the need to identify general factors that influence the success or failure of biocontrol in plant pathology, and performed a quantitative synthesis of published research to determine the overall effectiveness of biocontrol in relation to biological factors (e.g., BCA type) and application factors (e.g., number of treatments). Our results provide further insight into understanding inconsistent results of studies conducted under different environmental conditions.

The equations developed in this work will enable researchers to predict with high accuracy the relative control of a BCA for any combination of T, RH, and time before infection by *B. cinerea* conidia. To our knowledge, no similar equations have been previously reported. These equations may help farmers select which BCA to use for a specific treatment based on weather conditions at the time of application and in the following days, and based on the predicted time of *B. cinerea* infection. An example is provided in **Figure 5**; when temperature and relative humidity are known for a vineyard at the time of BCA treatment and in the following days, which BCA would better express its potential to control BBR can be easily determined. Similarly, when a prediction of infection by *B. cinerea* is available, the BCA that would express higher potential control at that time can be determined (see **Figure 4**).

Temperature and humidity conditions can be recorded in the vineyard by using calibrated weather sensors that provide the data to disease prediction models (Sanna et al., 2018); the use of weather forecasts in predictive systems for disease management is also common (Bourke, 1970; Gent et al., 2013; Olatinwo and Hoogenboom, 2014), thanks to a progressive increase in their accuracy (Bauer et al., 2015) and to the availability of methods for refining forecast data using point observations (Leblanc, 2019). A mechanistic, weather-driven model for accurate prediction of *B. cinerea* infection risk in vineyards has been developed and validated (González-Domínguez et al., 2015; Fedele et al., 2020b). Therefore, equations developed in this work have the potential to be used in practice, but their reliability under the fluctuating temperature and humidity conditions of vineyards remains to be evaluated and their utility should be verified with field experiments (Xu, 1996; Rossi et al., 2002; Legler et al., 2012). Another limitation of this study is that it provides data only for BBR resulting from direct infection of berries by *B. cinerea* conidia. As noted in the *Introduction*, BBR can also result from latent infections established during flowering and from berry-to-berry infection caused by mycelium originating from previously infected berries within the cluster.

In this research, we did not consider the differences between the environmental requirements of *B. cinerea* and BCAs, because the berries were incubated at optimal conditions for *B. cinerea* after the *B. cinerea* inoculation. *B. cinerea* is active at a wide range of temperatures and has an optimum range of 20–25°C (Ciliberti et al., 2015). The temperature range for the optimal growth for most of the BCAs tested for *B. cinerea* control is 18–32°C (Fedele et al., 2020c), i.e., the ranges overlap but only partially. Consider, for example, that the optimum temperature range is 23–28°C for *Aureobasidium* spp. (Lima et al., 1997) and 25–30°C for *Bacillus* spp. (Calvo et al., 2017). Given the rapid and abundant sporulation of *B. cinerea* on berries at sub-optimal temperatures (Ciliberti et al., 2016), neither *Aureobasidium* spp. nor *Bacillus* spp. may be able to prevent the rapid colonization of berries by *B. cinerea* at temperatures below 23°C. This concern was considered by Fedele et al. (2020c) by using an environmental niche approach. Environmental niches, defined as the environmental conditions necessary for the presence of a species and for the maintenance of its population (Chesson et al., 2001), could be useful for studying the temperature and humidity conditions under which a BCA prevails over *B. cinerea*, and for defining the extent of environmental niche sharing between a BCA and the target pathogen. The two approaches–i.e., equations developed in the current study and the environmental niche charts from Fedele et al. (2020c)—could be usefully combined. The equations could be used to evaluate the effect of environmental conditions occurring between the time of BCA application and *B. cinerea* infection, and the environmental niche charts could be used to evaluate the environmental effects when both the BCA and the pathogen are present and interacting on the host surface.

## Data Availability Statement

The raw data supporting the conclusions of this article will be made available by the authors, without undue reservation.

## Author Contributions

VR mainly contributed to the conception and the design of the study. GF and CB carried out the experiments. VR contributed to the analysis of results. GF and VR wrote the manuscript. All authors contributed to the article and approved the submitted version.

## Conflict of Interest

The authors declare that the research was conducted in the absence of any commercial or financial relationships that could be construed as a potential conflict of interest.
